# Invasive Brain-Computer Interfaces: A Critical Assessment of Current Developments and Future Prospects

**DOI:** 10.2196/60151

**Published:** 2024-07-19

**Authors:** Pieter Kubben

**Affiliations:** 1 Department of Neurosurgery Maastricht University Medical Center Maastricht Netherlands

**Keywords:** brain computer interfacing, neurotechnology, brain-computer, interfacing, interface, interfaces, invasive, human-machine, human-computer, BCI, BCIs, brain-computer interface, neuroscience, technology, digital health, brain, machine learning, artificial intelligence, AI, ethics, innovation, policy, innovation, mHealth, mobile health

## Abstract

Invasive brain-computer interfaces (BCIs) are gaining attention for their transformative potential in human-machine interaction. These devices, which connect directly to the brain, could revolutionize medical therapies and augmentative technologies. This viewpoint examines recent advancements, weighs benefits against risks, and explores ethical and regulatory considerations for the future of invasive BCIs.

## Perspective

Invasive brain-computer interfaces (BCIs) have recently attracted significant attention due to their potential to revolutionize the interaction between humans and machines. By directly interfacing with the brain, these devices offer profound implications for medical therapies and augmentative technologies. This viewpoint discusses the latest advancements, evaluates the benefits against the potential risks, and considers the ethical and regulatory landscapes shaping the future of invasive BCIs.

BCIs that involve invasive techniques, such as surgically implanted electrodes, are not new concepts but have seen rapid development in recent years. These devices provide a direct pathway for decoding and modulating neural activity, thereby offering unprecedented opportunities for patients with severe neurological deficits to interact with their environments in ways previously deemed unfeasible.

The progress in microfabrication technology, neural decoding algorithms, and materials science has substantially increased the capabilities of invasive BCIs. Modern electrodes can now be manufactured at scales small enough to minimize damage while maintaining high fidelity in signal recording. Techniques like endovascular BCI approaches propose minimally invasive methods to place electrodes closer to relevant neural tissues without traditional open-brain surgery [1]. Their clinical potential still has to be demonstrated.

Invasive BCIs are primarily aimed at restoring lost functions such as mobility, speech, and even cognitive faculties in patients with disabilities resulting from conditions like stroke, spinal cord injuries, and neurodegenerative diseases. For example, devices have been developed to enable individuals with paralysis to control robotic limbs or computer cursors with their thoughts alone [2,3]. Beyond therapeutic applications, there is also exploratory research into the use of BCIs for enhancing human memory and cognitive speed, suggesting a potential expansion into augmentation uses in the future [4].

The capability of BCIs to read and potentially write to the human brain raises significant ethical questions. Issues such as consent, autonomy, and the potential for influencing voluntary choices or privacy violations are of paramount concern. The privacy of neural data, akin to digital and genetic information, requires stringent safeguards to prevent unauthorized access and misuse [4-6]. To some extent, such concerns are already applicable to, for example, deep brain stimulation devices, but BCIs will take them to the next level.

The implantation of BCI devices involves invasive procedures that carry inherent risks such as infection, inflammation, and the potential for long-term immune responses. Moreover, the permanency of these implants poses challenges in device maintenance and updates, complicating their management over a patient’s lifetime [5,7]. Regulatory bodies are currently grappling with these issues, striving to develop guidelines that ensure patient safety without stifling innovation. Another area of concern is postexplantation care, in particular in research settings. For example, when study participation results in improved functioning, ethical concerns will arise when the study concludes and participation must stop.

As BCIs advance, they could significantly alter many aspects of society, from health care to employment, potentially leading to new forms of inequality. Access to and control of such powerful technologies could exacerbate social divides if not carefully managed. Public discussion and policy development must therefore keep pace with technological advancements to address these societal impacts comprehensively.

## Conclusion

Invasive BCIs hold tremendous promise for transforming lives, particularly for those with severe disabilities. However, the rapid pace of development in this field necessitates careful consideration of the ethical, safety, and societal issues that accompany such transformative technologies. Balancing innovation with responsible development will be key to realizing the full potential of BCIs while minimizing potential harms.

## Abbreviations

BCI: brain-computer interface
